# Correlates of Viral Suppression in Adolescents With HIV Taking Antiretroviral Therapy in Mozambique: Results of a Multicenter Cross-Sectional Survey

**DOI:** 10.1016/j.jadohealth.2026.02.023

**Published:** 2026-05-05

**Authors:** Joana Falcao, Elaine J. Abrams, Frédérique Chammartin, Aleny Couto, Allison Zerbe, Eduarda Pimentel De Gusmao, Kelvin Manuel, Niklaus D. Labhardt

**Affiliations:** a ICAP at Columbia University, Mailman School of Public Health, Maputo, Mozambique; b Clinical Epidemiology, Department of Clinical Research, University and University Hospital Basel, Basel, Switzerland; c ICAP at Columbia University, Mailman School of Public Health, New York, New York; d Department of Epidemiology, Mailman School of Public Health, Columbia University, New York, New York; e Department of Pediatrics, Vagelos College of Physicians and Surgeons, Columbia University, New York, New York; f National STI, HIV/AIDS Control Program, Maputo, Mozambique

**Keywords:** HIV, ART, Adolescents, HIV viral suppression, Mozambique

## Abstract

**Purpose:**

Adolescents living with HIV (ALHIV) have poorer outcomes on antiretroviral therapy (ART) than adults. We assessed correlates of viral suppression (VS) among ALHIV, aged 15–19 years, in Mozambique.

**Methods:**

This is a nested analysis within the CombinADO trial (NCT04930367), a cluster randomized trial assessing the effectiveness of a multilevel health services intervention to improve VS (HIV RNA <50 copies/mL) among ALHIV. After 12 months of intervention implementation, ALHIV at 12 health facilities were approached for enrolment into a postintervention assessment that included surveys and a blood draw for viral load (VL) measurement. Multivariable logistic regression reporting adjusted odds ratios (aORs) was used to quantify the association of different factors with VS. Data were collected between November 2022 and July 2023.

**Results:**

Of 544 ALHIV 15–19 years (72% female) included in this analysis, 300 (55%) had VS. ALHIV who understood their latest VL result were more likely to have VS (aOR = 1.59, 95% confidence interval [CI] 1.05–2.42). VS was lower among ALHIV who had moderate and high levels of internalized stigma (aOR = .45, 95% CI .21–.96 and aOR = 0.38, 95% CI .17–.88, respectively).

**Discussion:**

Reducing internalized stigma and improving understanding of VL results may contribute to improving VS among ALHIV in Mozambique. These findings underscore the urgent need for adolescent tailored HIV care in Mozambique.

Adolescence is a dynamic period spanning from the onset of puberty to adulthood and involves the acquisition of physical, cognitive, emotional, social, and economic resources that form the foundation for later life, health, and well-being [1–3]. The last decades of research on adolescent brain development have made important advancements in explaining the neural processes and alterations that support the profound changes observed during this period, including physical maturation but also development of social behavioral skills and risk-taking behaviors [4]. However, despite the recognized importance of this developmental stage, adolescents still have many unmet needs for health care often associated with substance use, mental health, reproductive health, and communicable diseases including HIV, among others.

Despite the tremendous gains and advances in curbing the HIV and AIDS epidemic globally, progress has stalled for children and adolescents with HIV across the testing and treatment cascade [5]. In 2023, of the estimated 1.55 million adolescents aged 10–19 living with HIV (ALHIV) worldwide, only 65% received antiretroviral therapy (ART) [6]. Moreover, for those on ART, rates of HIV viral suppression, which is critical to improve and preserve individual health, avert sexual and perinatal transmission, are substantially poorer than adults [6–8]. The WHO Africa Region is home to 82% of all ALHIV. AIDS continues to be the leading cause of death in this population, with adolescents’ girls and young women being disproportionately affected as their risk of HIV acquisition is up to 6-fold higher than boys and young men in the same age group [9].

A major reason for poor virologic outcomes among ALHIV is suboptimal adherence to ART. Numerous studies report barriers to ART adherence among ALHIV are multiple and multifaceted, acting at individual, interpersonal, and community level. In recent years, important correlates for nonadherence and viral suppression in Africa among ALHIV have been found to be stigma, ART side effects, lack of social support from caregivers, peers, the health system, and school environments, forgetfulness, delays in HIV disclosure, and economic constraints [10–12]. As children born with HIV transition into adolescence—while new HIV acquisitions continue to occur in this age group—the management of HIV across adolescence poses substantial challenges for health services, families, and adolescents themselves [13,14].

Mozambique, located in southern Africa, remains among the countries most heavily affected by HIV [15]. Mozambique has an HIV prevalence of 11.5% among adults and 4.3% among 15–24 years old [15]. In 2024, of the over 2.5 million people living with HIV in the country, 12% were 15–24-years old and of the 92,000 new acquisitions, 40% occurred among 15–24 years old [15]. In 2021, only 49% of ALHIV were taking ART and only 35.8% had viral suppression <1,000 copies/mL [16]. Moreover, pervasive poverty, affecting nearly three quarters of the population and limiting access to health and education, disproportionately impacts young people and contributes to poor health outcomes [17,18]. Data on viral suppression rates and its correlates among ALHIV in Mozambique are scarce. Knowing factors associated with viral suppression may inform future approaches to tailor ART care to ALHIV and consequently improve outcomes. In this study, we assess potential correlates of viral suppression among ALHIV aged between 15 and 19 years who access ART care in Nampula province, Mozambique.

## Methods

### Study setting and design

This is a nested cross-sectional analysis of data generated by the CombinADO trial (NCT04930367) conducted in Nampula, Mozambique. The CombinADO study was a cluster randomized trial designed to assess the effectiveness of a multilevel health services intervention (the CombinADO strategy) versus optimized standard of care, on viral suppression among ALHIV, aged 10–24 years, in 12 health facilities of seven districts of Nampula Province. Nampula province, located in the northern part of the country, is the most populated province, with 5.8 million people. The province has an estimated HIV prevalence estimated of 10% for adults and 4.6% for 15–24 years old [16]. In summary, the CombinADO intervention consisted of an empathy-building community campaign (billboards, radio ads), youth-friendly clinic experience (treatment and self-reflection toolkits, motivational video/wall, mental health screening, peer support) and ALHIV/caregiver support groups. Further details on CombinADO study are given elsewhere [19]. The present nested analysis focuses on adolescent CombinADO participants, aged 15–19 years. Data were collected between November 2022 and July 2023.

### Participants and procedures

The inclusion criteria for the CombinADO study were being registered in care and taking ART at one of 12 CombinADO study sites; being aware of one’s own HIV status; being between 10 and 24 years and agreeing to all study procedures. Participants were excluded if they had an acute medical condition requiring immediate medical care; and or any other condition, that, in the opinion of the study team, precluded informed consent.

After 12 months of intervention implementation CombinADO participants underwent a postintervention assessment, which included administration of a survey and a venous blood draw for viral load (VL) measurement. The survey was administered in Portuguese or Makhuwa via electronic tablets and responses were entered into SurveyCTO. Blood samples were sent to the Molecular Biology Laboratory at the Central Hospital in Nampula, where VL testing was performed on the COBAS AmpliPrep/COBAS TaqMan. VL results were reviewed and returned to study sites to inform onward care. Additionally, any participants completing the survey who screened positive for mental health problems, sexual or physical abuse, and food insecurity were referred to appropriate care.

### Data

Viral suppression, used as indicator for adequate adherence, was defined as HIV RNA <50 copies/mL. For this analysis, we selected a subset of variables and scales from the CombinADO postintervention survey based on previously reported associations with adherence or viral suppression in the literature and guided by a social ecological model [20], which hypothesizes that factors at individual, interpersonal, and community levels are associated with HIV viral suppression. The variables that were selected for our model are described below. The detailed data collection instrument is provided in the appendix 3. At individual level variables included age (15–17/18–19 years), gender (male/female), perinatal HIV acquisition (“yes” if adolescents reported initiating ART before the age of 15 or reported never having had sexual intercourse, otherwise “no”), any reported side effects attributed to ART including headaches, nausea, stomach aches, changes in appetite or weight, dizziness or others (no/yes), last VL result understanding (never received a VL result/VL result received but no understanding/VL result received and understanding), internalized stigma (none/moderate/high, measured using an adaptation of the HIV Stigma Scale for South African Adolescents Living with HIV [21], depression (minimal or mild depression (0–9) (moderate or moderately severe or severe (0–9 vs. 10–27), according to the patient health questionnaire 9 [22], anxiety (minimal or mild/moderate or severe (0–9 vs. 10–24), according to the general anxiety disorder questionnaire [23] and ART regimen (dolutegravir-based regimen/other). At interpersonal level variables included relationship status (not in a relationship/in a relationship and not married/married or cohabiting), disclosure of HIV status to caregiver (a family member or a partner or spouse) (no/yes), disclosure of HIV status to friends (no/yes), exposure to violence from a relative (no/yes if ever been slapped, punished, or beaten by a relative or ever been sworn at, insulted, or humiliated by a relative), exposure to sexual violence (no/yes if ever been touched on sexual or private parts by an adult or, someone older without consent, or ever been forced or pressured to have sex at a time when were unable to refuse), HIV associated bullying (no vs. yes if friends/partners were lost after HIV status disclosure, or were teased because of HIV status, or were avoided being touched after others knowing HIV status, or received bad reaction when others found out HIV status). At community level, variables included economic status of the household (lowest or moderate/highest according to a standardized asset score built from combination of presence or absence of electricity, water, and toilet), household food insecurity in the last 4 weeks (secure or mildly insecure/moderately/severely unsecure according to the Household Food Insecurity Access Scale Questionnaire [24], enrolled in school (no/yes), and receiving care at either an intervention site or a control site in the CombinADO trial (control/intervention).

### Ethics statement

The CombinADO study protocol was approved by the Columbia University Irving Medical Center Institutional Review Board and the National Bioethics Committee for Health in Mozambique. The present analysis constitutes a nested analysis of the CombinADO study and is fully covered by the original ethical approval. The CombinADO protocol and its informed consent forms explicitly included the cross-sectional survey analyzed in this manuscript. All participants provided written, dated informed consent; for adolescents younger than 18 years, written assent was obtained from the adolescent together with written informed consent from a caregiver. All study personnel were trained in Good Clinical Practice. Interviews were conducted in private settings. Data used for the present analysis were pseudonymized, with personal identifiers removed prior to analysis, and access restricted to authorized study staff. Data were stored and managed in accordance with institutional data protection and confidentiality policies. Participants received US $7 to compensate for transportation costs and time.

#### Statistical analysis.

Participant characteristics are summarized as absolute and relative frequencies. We developed a multivariable logistic regression model to assess associations between multiple variables and the risk of viral suppression. We report adjusted odds ratios (aORs) with 95% confidence intervals (CIs), together with crude odds ratio from univariate analysis. All variables from the univariate analysis were added to the multivariable analysis to estimate correlates that might independently be associated with viral suppression. Our aim was to understand how individuals, interpersonal, and society level factors a priori chosen based on theoretical and empirical evidence jointly operate. No variable selection was performed as we were not limited by the sample size. Multicollinearity was assessed using the variance inflation factor [25], with no significant multicollinearity being found. Robustness of our analysis with regards to viral suppression definition was assessed within a sensitivity analysis that modified the threshold for viral suppression to <200 copies/mL. This threshold was chosen because it is recommended in the Guidelines for the Use of Antiretroviral Agents in Adults and Adolescents with HIV issued by the National Institute of Health, which define viral suppression as <200 copies/mL to exclude most cases of apparent viraemia attributable to transient VL blips or assay variability and as the threshold below which HIV is considered non-transmissible [26]. All analyses were performed using Stata version 18 for MacOS [27].

## Results

Between November 2022 and July 2023, 1,715 participants aged 10–24 years enrolled in the CombinADO trial at the 12 study sites. Overall, 544 were between 15–19 years old and included in this nested analysis. Study participant characteristics, overall, and by viral suppression (VL < 50 copies/mL) status, are presented in Table 1. Three hundred (55.2%) had viral suppression <50 copies/mL. Looking at individual level factors, most of the study population was female (71.3%), and nearly half (47.2%) were classified as having perinatal infection. Almost all participants were taking a dolutegravir-based regimen (98.7%), and 44.9% reported experiencing at least one side effect from their current ART regimen. When asked about their understanding of their latest VL result, 30.9% reported not understanding the result, 53.5% understanding the result, and 15.6% reported never having done a VL test. Moderate and high internalized stigma was reported by 59.4% and 32.5%, respectively. A total of 24.6% screened positive for moderately severe/severe depression and a total of 23.7% for moderate/severe anxiety. Looking at interpersonal factors 80.7% had disclosed HIV status to a caregiver (a family member or a partner or spouse) and 17.3% had disclosed their HIV status to any friend. Approximately half (47.1%) were not in a relationship, 33.5% were in a relationship but not married and 19.5% were married or cohabiting with a partner. Regarding experiences of different types of violence, 29.6% reported having experienced HIV-associated bullying; 19.3% reported sexual violence; and 41.4% reported emotional or physical violence from a relative. Regarding community factors, 72.2% were from lowest/moderate economic status, 61.0% suffered from severe food insecurity, and 78.7% were currently in school.

Table 2 shows univariable and multivariable analyses of the association between individual, interpersonal, and community variables with viral suppression. ALHIV who reported understanding their latest VL result had significantly higher odds of viral suppression than ALHIV who did not understand their VL result (aOR = 1.59, 95% CI 1.05–2.42). On the other hand, ALHIV reporting moderate and high levels of internalized stigma had lower odds of viral suppression than those without internalized stigma (aOR = .45, 95% CI .21–.96 and aOR = .38, 95% CI .17–.88, respectively). Adjusted effect estimates from multivariable models were consistent with unadjusted estimates and none of the independent variables included in our multivariate analysis showed strong collinearity according to the variance inflation factor.

Using a less conservative threshold for viral suppression increased the overall percentage of ALHIV with viral suppression by 17% (55.2% and 71.5% viral suppression for thresholds < 50 and < 200 copies/mL, respectively). Moderate and high levels of internalized stigma remained significantly associated to lower odds of viral suppression than no internalized stigma (aOR = .36, 95% CI .14–.89 and aOR = .27, 95% CI .10–.74, respectively), while the effect of VL result understanding was no longer significant (more details on participants characteristics and regression model in appendices 1 and 2).

## Discussion

This cross-sectional study nested in the CombinADO trial assessed individual, interpersonal, and community correlates of HIV viral suppression among ALHIV aged between 15 and 19 years, receiving ART in 12 health facilities in Nampula, Mozambique. We identified internalized stigma and understating of VL result as important correlates of HIV viral suppression. In line with other studies assessing viral suppression among ALHIV, overall rates of suppression in our study were low [7,28,29].

Internalized stigma occurs when adolescents absorb negative messages and stereotypes about HIV creating a negative self-image [30]. Our study participants showed high levels of internalized stigma, with more than half of the young people reporting at least a moderate level of internalized stigma and a third (32.5%) a high level of internalized stigma. High prevalence of stigma has been reported in previous studies with ALHIV [31–33]. In our study, moderate to high levels of internalized stigma were associated with lower likelihood of viral suppression. Accumulated evidence from both quantitative and qualitative studies in similar settings indicates that stigma—especially internalized stigma—and HIV-related discrimination are significant barriers to adolescents’ engagement with HIV services. These factors are linked to poor treatment adherence, lower chances of viral suppression, and worse mental health outcomes [32,34–39].

In our study, we found low levels of VL result understanding. A qualitative study with ALHIV, conducted in Zimbabwe, identified similar gaps in VL literacy [40]. ALHIV in Zimbabwe reported that VL was perceived as a procedure used by health care providers to monitor ART adherence but descriptions on how viral suppression can improve health and well-being and eliminate the risk of onward transmission were absent from participants’ narratives. The findings of our study indicate that the comprehension of the VL result was associated with an increased likelihood of viral suppression. Various studies have found health literacy to be associated with better health outcomes and adherence to various medical regimens. Even though health literacy is key for a healthy HIV self-management, it’s association with treatment adherence and viral suppression requires further investigation, particularly for the adolescent population [41–44].

Our study’s strengths come from it being a multicenter study with very few missing data and using a biologic marker, VL, as an objective indicator for ART adherence. However, limitations should be considered when interpreting the results. First, our study excluded individuals disengaged from care as being engaged in care and receiving treatment was part of the enrolment criteria of CombinADO. Second, our cross-sectional design does not allow deriving causation of the observed associations. Third, as this was a secondary data analysis, we did not collect variables specifically for our analytical purposes. Hence, we were limited in our choice of potential correlates for viral suppression. Future studies may benefit from including further variables such as family targeted adherence support, that is, on family support to the ALHIV.

Notwithstanding these limitations, our findings carry important implications. The characteristics of our adolescent population denoted multiple vulnerabilities: a large proportion of participants reported being exposed to emotional or physical violence from their relatives; others reported exposure to sexual violence, and a considerable number required further assessment for mental health problems. This accumulation of vulnerabilities is worrying, and further research should be conducted to assess to what extent it reflects the reality of the overall ALHIV population or even the general adolescent population in Mozambique. As of today, country-specific data on overall adolescent health and well-being is scarce and we did not find any data from Mozambique on the vulnerability data variables we explored. Enhancing the health of the adolescent population in Mozambique, which has over a third of its population aged between 10 and24 years [45], it is not only imperative but has the potential to engender what has been termed a “triple dividend”: namely, improvements in ALHIV health in the present, during their life course, and contributions to the health of future generations [46,47].

Study participants face concerning challenges related to internal stigma, and concomitantly a strong reluctance to disclose their status to friends (only 17% had disclosed their HIV status to at least one friend), who frequently serve as crucial sources of emotional and social support, particularly during adolescence. Consequently, the role of caregivers and relatives becomes increasingly pivotal in providing support for ALHIV on ART [48–52], including their engagement in stigma-reduction interventions. Caregivers of ALHIV may, however, need support themselves to cope with the stress of caring for an adolescent with a chronic and highly stigmatized condition [53–55]. Hence, HIV care for ALHIV should address the need for stigma-reduction interventions alongside supporting and improving caregiver engagement throughout the entire adolescence. Recent systematic reviews indicate that several interventions show promise in reducing HIV-related stigma and discrimination, including cognitive behavioral therapy, empowerment approaches, youth peer mentoring, community education, and client-centered counseling [56–59]. However, as of today, support for caregivers of adolescents living with HIV—and their sustained engagement in care across the adolescent developmental period—remains comparatively under-addressed in the adolescent HIV response.

Finally, the low level of VL result understanding and low rate of viral suppression among the ALHIV in shows an urgent need for HIV services to be responsive to the needs of young people. Effective ART support for adolescents in Mozambique may include more systematic measurements of VL alongside a personalized discussion of the results to identify and minimize the individual adherence challenges faced by each ALHIV. Existing peer interventions to support ALHIV in eastern and southern Africa which include a health literacy component such as the Zvandiri program in Zimbabwe or the Operation Triple Zero in Kenya can provide useful examples for the design of new programs for ALHIV in Mozambique [60].

### Conclusions

This study adds to the evidence on correlates of viral suppression among ALHIV. In the context of concerningly low rates of viral suppression in this population, adolescents experienced multiple intersecting vulnerabilities, with internalized stigma and limited understanding of VL results associated with lower odds of viral suppression. Furthermore, our results highlight the importance of caregiver engagement in HIV care for adolescents. Adolescent-responsive HIV programs should explicitly address these factors to improve treatment outcomes.

## Supplementary Material

1

2

3

## Figures and Tables

**Table 1:** Characteristics of adolescents living with HIV aged 15–19 years, by viral suppression status Nampula, Mozambique

Characteristics	Overall study population		Virally unsuppressed (≥50 copies/mL)		Virally supressed (<50 copies/mL)	
	n = 544		n = 244 (45.8%)		n = 300 (55.2%)	
	n	%	n	%	n	%
Age						
15–17 years old	256	47.1	123	50.4	133	44.3
18–19 years old	288	52.9	121	49.6	167	52.9
Gender						
Male	156	28.7	73	29.9	83	27.7
Female	388	71.3	171	70.1	217	72.3
Perinatal HIV infection						
No	287	52.8	121	49.6	166	55.3
Yes	257	47.2	123	50.4	134	44.7
ART regimen^a^						
Dolutegravir-based regimen	537	98.7	241	98.8	296	98.7
Reported side effects						
No	300	55.1	143	58.6	157	52.3
Yes	244	44.9	101	41.4	143	47.7
Internalized stigma						
None	44	8.1	14	5.7	30	10.0
Moderate	323	59.4	146	59.8	177	59.0
High	177	32.5	84	34.4	93	31.0
Depression						
Minimal/mild	410	57.4	186	76.2	224	74.7
Moderate/moderately severe/severe	134	24.6	58	23.8	76	25.3
Anxiety						
Minimal/mild	415	76.3	191	78.3	224	74.7
Moderate/severe	129	23.7	53	21.7	76	25.3
Relationship status						
Not in a relationship	256	47.1	125	51.2	131	43.7
In a relationship, not married	182	33.5	80	32.8	102	34.0
Married/cohabiting	106	19.5	39	16.0	67	22.3
Disclosure of HIV status to caregiver						
No	105	19.3	48	19.7	57	19.0
Yes	439	80.7	196	80.3	243	81.0
Disclosure of HIV status to friends						
No	450	82.7	205	84.0	245	81.7
Yes	94	17.3	39	16.0	55	18.3
Last viral load result understanding						
VL result not understood	168	30.9	83	34.0	85	28.3
VL result understood	291	53.5	113	46.3	178	59.3
Has never done a VL test	85	15.6	48	19.7	37	12.4
Exposure to HIV associated bullying						
No	383	70.4	175	71.7	208	69.3
At least one type of bullying	161	29.6	69	28.3	92	30.7
Exposure to violence from a relative						
No	319	58.6	134	54.9	185	61.7
At least one type of violence	225	41.4	110	45.1	115	38.3
Exposure to sexual violence						
No	439	80.7	202	82.8	237	79.0
At least one type of sexual violence	105	19.3	42	17.2	63	21.0
Economic condition						
Lowest	393	72.2	182	74.6	211	70.3
Moderate/highest	151	27.8	62	25.4	89	29.7
Food insecurity						
Food secure/mild food insecurity	121	22.2	51	20.9	70	23.3
Moderately food insecure	91	16.7	43	17.6	48	16
Severely food insecure	332	61.0	150	61.5	182	60.7
Currently in school						
No	116	21.3	48	19.7	68	22.7
Yes	428	78.7	196	80.3	232	77.3
CombinADO trial intervention group						
Enhanced SOC sites	316	58.1	138	56.6	178	59.3
Intervention sites	228	41.9	106	43.4	122	40.7

ART = antiretroviral therapy; VL = viral load. SOC = standard of care.

a Data were missing for seven participants. ART regimens were as follow: TDF+3TC+DTG; 3TC+AZT+DTG; ABC+3TC+DTG.

**Table 2:** Univariable and multivariable analysis of variables association with HIV RNA <50 copies/mL

Characteristics	Univariable regressions			Multivariable regression		
	OR	95% CI	*P* value	aOR	95% CI	*P* value
Age						
18–19 years (vs. 15–17 years)	1.28	.91–1.79	.158	1.21	.79–1.84	.380
Gender						
Female (vs. male)	1.12	.77–1.62	.564	1.02	.67–1.56	.928
Perinatal HIV infection						
Yes (vs. horizontal infection)	.79	.57–1.11	.182	.85	.53–1.38	.518
Reported side effects						
Yes (vs. no)	1.29	.92–1.81	.218	1.21	.84–1.75	.304
Last viral load result understanding						
VL result understood (vs. VL result not understood)	1.54	1.05–2.26	.028	1.59	**1.05–2.42**	**.030**
Has never done a VL test (vs. VL result not understood)	.75	.46–1.27	.289	.57	.32–1.01	.052
Internalized stigma						
Moderate (vs. none)	.57	.29–1.11	.096	.45	**.21–.96**	**.038**
High (vs. none)	.52	.26–1.04	.064	.38	**.17–.88**	**.024**
Depression						
Moderate/moderately severe/severe (vs. minimal/mild)	1.09	.73–1.61	.674	.84	.45–1.55	.578
Anxiety						
Moderate/severe (vs. minimal/mild)	1.22	.81–1.82	.325	1.17	.65–2.09	.608
Relationship status						
In a relationship, not married (vs. not in a relationship)	1.21	.83–1.78	.314	1.16	.76–1.71	.492
Married/cohabiting (vs. not in a relationship)	1.64	1.03–2.61	.037	1.70	.94–3.08	.082
Disclosure of HIV status to caregiver						
Yes (vs. no)	1.04	.68–1.60	.843	1.01	.63–1.63	.954
Disclosure of HIV status to friends						
Yes (vs. no)	1.18	.75–1.85	.471	.95	.578–1.58	.857
Exposure to HIV-associated bullying						
At least one type of bullying (vs. no)	1.12	.77–1.63	.544	1.09	.71–1.67	.685
Exposure to violence from a relative						
At least one type of violence (vs. no)	.76	.54–1.07	.112	.75	.51–1.09	.129
Exposure to sexual violence						
At least one type of violence (vs. no)	1.29	.83–1.97	.266	1.32	.81–2.13	.261
Economic condition						
Moderate/highest (vs. lowest)	1.24	.85–1.81	.271	1.14	.75–1.74	.537
Food insecurity						
Moderate (vs. none/mild insecurity)	.81	.47–1.41	.459	.94	.51–1.71	.832
Severe (vs. none/mild insecurity)	.88	.58–1.35	.566	.95	.94–1.55	.825
Currently in school						
Yes (vs. no)	.84	.55–1.27	.397	.97	.59–1.58	.888
CombinADO trial intervention group						
Intervention sites (vs. enhanced SOC sites)	.89	.63–1.26	.514	.75	.38–3.84	.144

Models include all 544 participants as no participant had missing values on the covariates included in the table.

CI = confidence interval; OR = odds ratio; aOR = adjusted odds ratio; VL = viral load.

Bold values indicate *p*-value statistically significant a 5%.
