# Supplementary material for: Correlates of Viral Suppression in Adolescents With HIV Taking Antiretroviral Therapy in Mozambique: Results of a Multicenter Cross-Sectional Survey
Source: J Adolesc Health. Author manuscript; Available in PMC 2026 Aug 26. (PMC13509033; doi:10.1016/j.jadohealth.2026.02.023)
Supplement: Supplementary file 1 [file NIHMS2191809-supplement-1.docx]

| **Appendix 1: Characteristics of Adolescents Living With HIV aged 15 to 19 years, by VS status (<200 copies/ ml), N= 544** | | | | |
| --- | --- | --- | --- | --- |
| **Characteristics** | **Virally unsupressed**  **(≥50 copies/mL)** | | **Virally supressed**  **(<50 copies/mL)** | |
|  | **n= 155 (28.5%)** | | **n=389 (72.5%)** | |
|  | **N** | **%** | **N** | **%** |
| **Age** |  |  |  |  |
| 15 - 17 | 86 | 55.5 | 170 | 43.7 |
| 18 - 19 | 69 | 44.5 | 219 | 56.3 |
| **Gender** |  |  |  |  |
| Male | 49 | 31.6 | 107 | 27.5 |
| Female | 106 | 68.4 | 282 | 72.5 |
| **Perinatal HIV infection** |  |  |  |  |
| No | 69 | 44.5 | 218 | 56.0 |
| Yes | 86 | 55.5 | 171 | 44.0 |
| **ART regimen *** |  |  |  |  |
| Dolutegravir based regimen | 154 | 99.4 | 383 | 98.5 |
| **Reported side effects** |  |  |  |  |
| No | 84 | 54.2 | 216 | 55.5 |
| Yes | 71 | 45.8 | 173 | 44.5 |
| **Last viral load result understanding** |  |  |  |  |
| VL result not understood | 83 | 34.0 | 85 | 28.3 |
| VL result understood | 113 | 46.3 | 178 | 59.3 |
| Has never done a VL test | 48 | 19.7 | 37 | 12.3 |
| **Internalized stigma** |  |  |  |  |
| None | 7 | 4.5 | 37 | 9.5 |
| Moderate | 90 | 58.1 | 233 | 59.9 |
| High | 58 | 37.4 | 119 | 30.6 |
| **Depression** |  |  |  |  |
| Minimal/ Mild | 111 | 71.6 | 299 | 76.9 |
| Moderate/Moderately severe/Severe | 44 | 28.4 | 90 | 23.1 |
| **Anxiety** |  |  |  |  |
| Minimal/ Mild | 117 | 75.5 | 298 | 76.6 |
| Moderate/Severe | 38 | 24.5 | 91 | 23.4 |
| **Relationship status** |  |  |  |  |
| Not in a relationship | 82 | 52.9 | 174 | 44.7 |
| In a relationship, not married | 47 | 30.3 | 135 | 34.7 |
| Married/ cohabiting | 26 | 16.8 | 80 | 20.6 |
| **Disclosure of HIV status to caregiver** |  |  |  |  |
| No | 27 | 17.4 | 78 | 20.0 |
| Yes | 128 | 82.6 | 311 | 80.0 |
| **Disclosure of HIV status to friends** |  |  |  |  |
| No | 127 | 81.9 | 323 | 83.0 |
| Yes | 28 | 18.1 | 66 | 17.0 |
| **Exposure to HIV associated bullying** |  |  |  |  |
| No | 107 | 69.0 | 276 | 71.0 |
| At least one type of bullying | 48 | 31.0 | 113 | 29.0 |
| **Exposure to violence from a relative** |  |  |  |  |
| No | 87 | 56.1 | 232 | 59.6 |
| At least one type of violence | 68 | 43.9 | 157 | 40.4 |
| **Exposure to sexual violence** |  |  |  |  |
| No | 130 | 83.9 | 309 | 79.4 |
| At least one type of sexual violence | 25 | 16.1 | 80 | 20.6 |
| **Economic condition** |  |  |  |  |
| Lowest | 110 | 71.0 | 283 | 72.8 |
| Moderate/Highest | 45 | 29.0 | 106 | 27.2 |
| **Food insecurity** |  |  |  |  |
| Food secure/mild food insecurity | 35 | 22.6 | 86 | 22.1 |
| Moderately food insecure | 24 | 15.5 | 67 | 17.2 |
| Severely food insecure | 96 | 61.9 | 236 | 60.7 |
| **Currently in school** |  |  |  |  |
| No | 27 | 17.4 | 89 | 22.9 |
| Yes | 128 | 82.6 | 300 | 77.1 |
| **CombinADO trial intervention group** |  |  |  |  |
| Enhanced SOC sites | 86 | 55.5 | 230 | 59.1 |
| Intervention sites | 69 | 44.5 | 159 | 40.9 |
| * Data was missing for seven participants. ART regimens were: TDF+3TC+DTG; 3TC+AZT+DTG; ABC+3TC+DTG | | | | |
