# Supplementary material for: Correlates of Viral Suppression in Adolescents With HIV Taking Antiretroviral Therapy in Mozambique: Results of a Multicenter Cross-Sectional Survey
Source: J Adolesc Health. Author manuscript; Available in PMC 2026 Aug 26. (PMC13509033; doi:10.1016/j.jadohealth.2026.02.023)
Supplement: Supplementary file 2 [file NIHMS2191809-supplement-2.docx]

| **Appendix 2: Multivariable analysis of variables association with HIV RNA <200 copies/mL** | | | | | | | |
| --- | --- | --- | --- | --- | --- | --- | --- |
|  | **Univariable regressions** | | | | **Multivariable regression** | | |
| **Characteristics** | **OR** | | **95% CI** | ***p*-Value** | **aOR** | **95% CI** | ***p*-Value** |
| **Age** | |  |  |  |  |  |  |
| 18-19 years (vs 15 - 17 years) | | 1.61 | 1.10 - 2.34 | 0.013 | 1.48 | 0.93 - 2.36 | 0.097 |
| **Gender** | |  |  |  |  |  |  |
| Female (vs male) | | 1.22 | 0.81 - 1.83 | 0.339 | 1.03 | 0.65 - 1.63 | 0.891 |
| **Perinatal HIV infection** | |  |  |  |  |  |  |
| Yes (vs horizontal infection) | | 0.63 | 0.43 - 0.92 | 0.015 | 0.73 | 0.43 - 1.24 | 0.242 |
| **Reported side effects** | |  |  |  |  |  |  |
| Yes (vs no) | | 0.95 | 0.65 - 1.38 | 0.778 | 0.97 | 0.65 - 1.45 | 0.875 |
| **Last viral load result understanding** | |  |  |  |  |  |  |
| VL result understood (vs VL result not understood) | | 1.12 | 0.73 - 1.70 | 0.603 | 1.27 | 0.80 - 2.01 | 0.314 |
| Has never done a VL test (vs VL result not understood) | | 1.02 | 0.57 - 1.80 | 0.954 | 0.74 | 0.40 - 1.82 | 0.355 |
| **Internalized stigma** | |  |  |  |  |  |  |
| Moderate (vs none) | | 0.49 | 0.21 - 1.14 | 0.097 | 0.36 | **0.14 - 0.89** | **0.027** |
| High (vs none) | | 0.39 | 0.16 - 0.92 | 0.032 | 0.27 | **0.10 - 0.74** | **0.010** |
| **Depression** | |  |  |  |  |  |  |
| Moderate/Moderately severe/Severe (vs minimal/mild) | | 0.76 | 0.50 - 1.16 | 0.200 | 0.65 | 0.33 - 1.27 | 0.210 |
| **Anxiety** | |  |  |  |  |  |  |
| Moderate/Severe (vs minimal/mild) | | 0.94 | 0.61 - 1.45 | 0.781 | 1.21 | 0.63 - 2.30 | 0.570 |
| **Relationship status** | |  |  |  |  |  |  |
| In a relationship, not married (vs not in a relationship) | | 1.35 | 0.89 - 2.07 | 0.161 | 1.16 | 0.72 - 1.86 | 0.538 |
| Married/cohabiting (vs not in a relationship) | | 1.45 | 0.87 - 2.43 | 0.157 | 0.94 | 0.49 - 1.82 | 0.865 |
| **Disclosure of HIV status to caregiver** | |  |  |  |  |  |  |
| Yes (vs no) | | 0.84 | 0.52 - 1.36 | 0.483 | 0.70 | 0.41 - 1.18 | 0.181 |
| **Disclosure of HIV status to friends** | |  |  |  |  |  |  |
| Yes (vs no) | | 0.93 | 0.57 - 1-51 | 0.76 | 0.91 | 0.53 - 1.57 | 0.744 |
| **Exposure to HIV associated bullying** | |  |  |  |  |  |  |
| At least one type of bullying (vs no) | | 0.91 | 0.61 - 1.37 | 0.658 | 0.99 | 0.63 - 1.58 | 0.988 |
| **Exposure to violence from a relative** | |  |  |  |  |  |  |
| At least one type of violence (vs no) | | 0.87 | 059 - 1.26 | 0.453 | 0.83 | 0.55 - 1.25 | 0.369 |
| **Exposure to sexual violence** | |  |  |  |  |  |  |
| At least one type of violence (vs no) | | 1.35 | 0.82 - 2.21 | 0.238 | 1.43 | 0.84 -2.46 | 0.191 |
| **Currently in school** | |  |  |  |  |  |  |
| Yes (vs no) | | 0.71 | 0.44 - 1.15 | 0.162 | 0.91 | 0.52 - 1.59 | 0.732 |
| **Economic condition** | |  |  |  |  |  |  |
| Moderate/ Highest (vs lowest) | | 0.92 | 0.61 - 1.38 | 0.675 | 0.95 | 0.60 - 1.50 | 0.820 |
| **Food insecurity** | |  |  |  |  |  |  |
| Moderate (vs none/mild insecurity) | | 1.14 | 0.62 - 2.09 | 0.682 | 1.31 | 0.68 - 2-53 | 0.427 |
| Severe (vs none/mild insecurity) | | 1.00 | 0.63 - 1.58 | 0.998 | 1.21 | 0.71 - 2.07 | 0.482 |
| **Currently in school** | |  |  |  |  |  |  |
| Yes (vs no) | | 0.71 | 0.44 - 1.15 | 0.162 | 0.91 | 0.52 - 1.59 | 0.732 |
| **CombinADO trial Intervention group** | |  |  |  |  |  |  |
| Intervention sites (vs enhanced SOC sites) | | 0.86 | 0.59 - 1.25 | 0.437 | 0.70 | 0.46 - 1.06 | 0.093 |
| * CI= confidence interval; OR= odds ratio; aOR= adjusted odds ratio. Models include all 544 participants as no participant had missing values on the covariates included in the table. | | | | | | | |
