# Supplementary material for: Correlates of Viral Suppression in Adolescents With HIV Taking Antiretroviral Therapy in Mozambique: Results of a Multicenter Cross-Sectional Survey
Source: J Adolesc Health. Author manuscript; Available in PMC 2026 Aug 26. (PMC13509033; doi:10.1016/j.jadohealth.2026.02.023)
Supplement: Supplementary file 3 [file NIHMS2191809-supplement-3.docx]

**Appendix 3: Questionnaire and response options used in the CombinADO trial**

| QUESTIONNAIRE | | |
| --- | --- | --- |
| PARTICIPANT ID NUMBER | |  |
| COMBINADO TRIAL INTERVENTION GROUP | |  |
| Q CODE | QUESTION | ANSWER |
| AGE | How old are you? | Age (years): _____________ |
| GENDER | What is your gender? | 1. Male 2. Female 3. Other (specify): ____________ |
| PERINATAL HIV INFECTION | How old were you the first time you had to take ART (treatment for HIV)? | Age (years) that ART was 1^st^ prescribed: ________ |
|  | Have you ever had vaginal, anal or oral sex?  *Check all that apply* | 1. Never had sex 2. Vaginal 3. Anal 4. Oral |
| REPORTED SIDE EFFECTS | Do you experience any of the following on your current ART regimen? | 1. Nausea 2. Vomiting 3. Stomach aches 4. Increased appetite 5. Decreased appetite 6. Weight gain 7. Weight loss 8. Headaches 9. Dizziness   Other, specify: __________ |
| INTERNALIZED STIGMA | *Now I am going to read a list of sentences.* Please tell me how frequent these thoughts/ situations for you are: | |
|  | Sometimes I feel I am not as good as the other adolescents my age because I have HIV. | 1 Never  2 Sometimes  3 Most of the time |
|  | Sometimes I feel I am a bad person because I have HIV. | 1 Never  2 Sometimes  3 Most of the time |
|  | Sometimes I feel ashamed I have HIV. | 1 Never  2 Sometimes  3 Most of the time |
|  | Sometimes I feel it is my fault I have HIV. | 1 Never  2 Sometimes  3 Most of the time |
|  | I work hard to keep my HIV a secret. | 1 Never  2 Sometimes  3 Most of the time |
| DEPRESSION  PHQ-9 modified for Adolescents (PHQ-A) | *How often have you been bothered by each of the following symptoms during the past two weeks?* | |
|  | Feeling down, depressed, irritable, or hopeless? | 1 Not at all  2 Several days  3 more than half days  4 Nearly every day |
|  | Little interest or pleasure in doing things? | 1 Not at all  2 Several days  3 more than half days  4 Nearly every day |
|  | Trouble falling asleep, staying asleep, or sleeping too much? | 1 Not at all  2 Several days  3 more than half days  4 Nearly every day |
|  | Poor appetite, weight loss, or overeating? | 1 Not at all  2 Several days  3 more than half days  4 Nearly every day |
|  | Feeling tired, or having little energy? | 1 Not at all  2 Several days  3 more than half days  4 Nearly every day |
|  | Feeling bad about yourself- or feeling that you are a failure, or that you have let yourself or your family down? | 1 Not at all  2 Several days  3 more than half days  4 Nearly every day |
|  | Trouble concentrating on things like school, work, reading, or watching tv? | 1 Not at all  2 Several days  3 more than half days  4 Nearly every day |
|  | Moving or speaking so slowly that other people could have noticed?  Or the opposite – being so fidgety or restless that you were moving around a lot more than usual? | 1 Not at all  2 Several days  3 more than half days  4 Nearly every day |
|  | Thoughts that you would be better off dead, or of hurting yourself in some way? | 1 Not at all  2 Several days  3 more than half days  4 Nearly every day |
| ANXIETY  GAD- 7 | *Over the last 2 weeks, how often have you been bothered by the following problems?* | |
|  | Feeling nervous or anxious | 1 Not at all  2 Several days  3 more than half days  4 Nearly every day |
|  | Not being able to stop or control worrying | 1 Not at all  2 Several days  3 more than half days  4 Nearly every day |
|  | Worrying too much about different things | 1 Not at all  2 Several days  3 more than half days  4 Nearly every day |
|  | Trouble relaxing | 1 Not at all  2 Several days  3 more than half days  4 Nearly every day |
|  | Being so restless it’s hard to keep still | 1 Not at all  2 Several days  3 more than half days  4 Nearly every day |
|  | Becoming easily annoyed or irritable | 1 Not at all  2 Several days  3 more than half days  4 Nearly every day |
|  | Feeling afraid as if something awful might happen | 1 Not at all  2 Several days  3 more than half days  4 Nearly every day |
| RELATIONSHIP STATUS | Are you currently in a romantic relationship? | 1 Yes 2 No |
|  | How would you describe your current relationship status? | 1. Married and living together 2. Married but not living together 3. Not married but living together 4. Not married and not living together 5. Not in a relationship |
| DISCLOSURE OF HIV STATUS TO CAREGIVER | Does your primary caregiver know that you have HIV? | 1. Yes 2. No   3 I don’t know |
| DISCLOSURE OF HIV STATUS TO FRIENDS | How many of your friends know that you are HIV positive? | 1. None 2. Some 3. Almost all |
| LAST VIRAL LOAD RESULT UNDERSTANDING | What was the result of your most recent viral load? | 1. It was low 2. It was undetectable 3. It was high 4. I don’t know/ I don’t remember 5. I haven’t done a VL test |
|  | Do you feel like you understand what your viral load test result means? | 1. Not at all 2. Very little 3. Fairly well 4. Very well 5. I haven’t done a VL test |
| EXPOSURE TO HIV ASSOCIATED BULLYING | Have you ever lost a friend/partner after disclosing to them your HIV status? | 1 No  2 Yes |
|  | Have you ever been teased because of your HIV status? | 1 No  2 Yes |
|  | Has anyone ever avoid touching you once they knew your HIV status? | 1 No  2 Yes |
|  | Has anyone ever reacted badly in anyway after discovering your HIV status? | 1 No  2 Yes |
| EXPOSURE TO VIOLENCE FROM A RELATIVE | Have you ever been slapped, punished, or beaten by a relative? | 1 No  2 Yes |
|  | Have you ever been sworn at, insulted, or humiliated by a relative? | 1 No  2 Yes |
| EXPOSURE TO SEXUAL VIOLENCE | Have you ever been touched on sexual or private parts by an adult or, someone older without consent? | 1 No  2 Yes |
|  | Have you ever been forced or pressured to have sex at a time when were unable to refuse? | 1 No  2 Yes |
| ECONOMIC CONDITION | Does your household das the following: |  |
|  | A toilet inside | 1 No  2 Yes |
|  | Running water inside | 1 No  2 Yes |
|  | Electricity inside | 1 No  2 Yes |
| FOOD INSECURITY  Household Food Insecurity Access Scale (HFIAS) | For the last four weeks: |  |
|  | In the past four weeks, did you worry that your household would not have enough food? | 1 No  2 Rarely (once or twice)  3 Sometimes (3 to 10 times)  4 Often (more than 10 times) |
|  | In the past four weeks were you or any household member not able to eat the kinds of foods you preferred because of a lack of resources (money)? | 1 No  2 Rarely (once or twice)  3 Sometimes (3 to 10 times)  4 Often (more than 10 times) |
|  | In the past four weeks did you or any household member have to eat a limited variety of foods due to a lack of resources (money)? | 1 No  2 Rarely (once or twice)  3 Sometimes (3 to 10 times)  4 Often (more than 10 times) |
|  | In the past four weeks, did you or any household member have to eat some foods that you really did not want to eat because of a lack of resources (money) to obtain other types of food? | 1 No  2 Rarely (once or twice)  3 Sometimes (3 to 10 times)  4 Often (more than 10 times) |
|  | In the past four weeks, did you or any household member have to eat a smaller meal than you felt you needed because there was not enough food? | 1 No  2 Rarely (once or twice)  3 Sometimes (3 to 10 times)  4 Often (more than 10 times) |
|  | In the past four weeks, did you or any household member have to eat fewer meals in a day because there was not enough food? | 1 No  2 Rarely (once or twice)  3 Sometimes (3 to 10 times)  4 Often (more than 10 times) |
|  | In the past four weeks, was there ever no food to eat of any kind in your household because of lack of resources (money) to get food? | 1 No  2 Rarely (once or twice)  3 Sometimes (3 to 10 times)  4 Often (more than 10 times) |
|  | In the past four weeks, did you or any household member go to sleep at night hungry because there was not enough food? | 1 No  2 Rarely (once or twice)  3 Sometimes (3 to 10 times)  4 Often (more than 10 times) |
|  | In the past four weeks, did you or any household member go a whole day and night without eating anything because there was not enough food? | 1 No  2 Rarely (once or twice)  3 Sometimes (3 to 10 times)  4 Often (more than 10 times) |
|  | In the past week, did you or any household member eat a cooked meal less than once a day? | 1 No  2 Rarely (once or twice)  3 Sometimes (3 to 10 times)  4 Often (more than 10 times) |
| CURRENTLY IN SCHOOL | What level of school do you currently go to? | 1. Primary 2. Secondary 3. Technical or vocational school 4. University, college or another tertiary institutional 5. I am not in school |
